# Spatial-Temporal Graph Convolutional Framework for Yoga Action Recognition and Grading

**DOI:** 10.1155/2022/7500525

**Published:** 2022-03-29

**Authors:** Shu Wang

**Affiliations:** School of Physical Education, Inner Mongolia Minzu University, Tongliao, Inner Mongolia 028000, China

## Abstract

The rapid development of the Internet has changed our lives. Many people gradually like online video yoga teaching. However, yoga beginners cannot master the standard yoga poses just by learning through videos, and high yoga poses can bring great damage or even disability to the body if they are not standard. To address this problem, we propose a yoga action recognition and grading system based on spatial-temporal graph convolutional neural network. Firstly, we capture yoga movement data using a depth camera. Then we label the yoga exercise videos frame by frame using long short-term memory network; then we extract the skeletal joint point features sequentially using graph convolution; then we arrange each video frame from spatial-temporal dimension and correlate the joint points in each frame and neighboring frames with spatial-temporal information to obtain the connection between joints. Finally, the identified yoga movements are predicted and graded. Experiment proves that our method can accurately identify and classify yoga poses; it also can identify whether yoga poses are standard or not and give feedback to yogis in time to prevent body damage caused by nonstandard poses.

## 1. Introduction

Yoga has become a very trendy fitness exercise in today's life. But yoga is much more than just a fitness exercise. Yoga is a physical and mental discipline that combines art, science, and philosophy. Yoga can help people regulate their breathing, keep their bodies healthy, and also calm their moods. In today's highly developed Internet, according to incomplete statistics, yoga has become the preferred fitness exercise for 300 million people [1]. As a scientific exercise, yoga encompasses breath control exercises, body stretching exercises, and mind cleansing [2]. Yoga originally originated in ancient India, then spread to the West, where it became a mainstream Western fitness modality, and then eventually spread globally with the Internet, becoming one of the most popular exercise cultures worldwide [3]. According to a joint UK and US survey, the demographic profile of the yoga training population found in the demographics indicates that women are the main enthusiasts of the sport, accounting for 85% of the total number of yoga practitioners [4–6].

Numerous studies have proven that yoga exercises are beneficial to the human body. There is also a large amount of research in rehabilitation on how to make yoga training work better for patients in their recovery process. This is one of the reasons why yoga has become a favorite exercise for many people [7]. In addition, research has proven that yoga has a complementary healing effect in the direction of eating disorders; it can modify the patient's eating habits and keep diet [8]. In the interviews of yoga practitioners, it was learned that yoga gave them a positive and subjective life experience, making them healthier and living an optimistic life. There were significant improvements in self-care, self-activity, life comfort, and dwelling senses [9–11]. In fact, most of the experience that yoga brings to people comes from the yoga instructor. The instructor, as the guide of yoga, influences the yoga student in an invisible way with his or her philosophy of teaching, teaching environment, outlook on life, values, and demonstration of yoga effectiveness [12].

Although some researchers have demonstrated that yoga can be practiced without differentiating between “traditional” and “authentic” issues [13], most people currently prefer modern yoga. Modern yoga is simpler and less demanding in terms of postural alignment and breathing exercises [14]. This is one of the reasons why modern yoga has turned into a healthy exercise for young and old alike. However, due to the overall economic development, yoga has gradually become commercialized. With the commercialization, the expression of yoga has become diversified and more and more people have become attracted to yoga. In our literature research, we found that yoga is becoming a synonym for young, beautiful, and hot women [15]. Yoga can be found in various fashion magazines and shows yoga poses that have a certain ornamental quality and at the same time these poses are difficult in the eyes of professionals. For ordinary people, they are more attracted by the ornamental poses of yoga, but these poses are risky for them. Commerce has made yoga idealized in order to facilitate promotion and thus attract consumers [16]. However, the commercialization of yoga is also a double-edged sword. Consumers are likely to cause irreversible damage to their bodies in the process of blindly imitating yoga poses due to the unknown nature of the poses, which is a potential risk in yoga training.

Traditionally, yoga is taught face-to-face, with the yoga instructor instructing in person whether the yoga poses are standard or not. This kind of teaching can make yoga students have a more direct feeling of standard yoga movements. However, with the advent of the 5G era and the rapid development of short videos, short video platform bloggers often adopt online teaching methods to teach yoga poses in order to attract fans. This is also the way most people learn yoga at present. Most people choose to watch videos while imitating to achieve the purpose of learning yoga. However, most people do not have professional yoga equipment and props, and they are not clear enough about the standard yoga postures. Blindly imitating the yoga postures in the videos has a great risk of physical injury. To solve this problem, in our work, we propose to use real-time posture detection technology to detect posture movements of yoga students and then use deep learning algorithms to grade and match yoga movements. A reference movement is given to the yoga students, and for the nonstandard movements, the yoga students are prompted in time to prevent the occurrence of physical injuries. In the specific experiment, we use the deep camera to capture the training postures of yoga students and decompose the postures to understand the yoga movements from the computer level. The postures are then compared with a standard database to verify whether the postures are standardized and to give feedback to the yoga students. Experiments show that the method proposed in our research can provide effective feedback to yoga trainees on the grading of yoga poses. The contributions of this paper can be summarized as follows.

The rest of this paper is organized in the following manner.  Section 2 discusses the work related to deep camera and action recognition.  Section 3 introduces the skeleton recognition principle of graph convolution, then introduces the residual unit and multistream input structure, and finally introduces the optimization principle of the partial perception framework.  Section 4 reports the experimental data collection, model training details, and analysis of experimental results. Finally, Section 5 concludes our research and reveals some further research work.

## 2. Related Work

The presentation of human motion postures in 3D space often requires the use of depth cameras. Information such as joint angles and skeletal space points can be deduced from the depth camera or the spatial position data of the human body [17]. Different poses can generate different skeletal contours, and to solve this problem, some researchers have proposed the idea of spatial segmentation, which takes an approximate mapping approach to define the location of spatial points for each segmented region. Literature [18] proposed a joint distribution method, which takes a bidirectional derivative approach to the mapping function. Literature [19] also uses the joint distribution rule, and unlike the former, the method adopts a Bayesian algorithm to obtain the image contour conditions. The final distribution of the image contour conditions will be mapped to the hybrid framework to obtain the spatial distribution features. Literature [17] additionally uses learning conditional distributions when learning features in the hybrid framework to obtain the image contour features more directly. In [20], to solve the image contour error problem caused by pose ambiguity, the researcher distributed three depth cameras into different angles to capture the human motion contour in all directions and obtained the skeletal spatial position from a standard dataset. In [21], the researcher used the SVM method to learn different pose features and perform pose prediction in the acquired 3D shape data. This proposed method links contours and 3D shapes but requires the support of large databases. For motion capture depth cameras, calibration of the depth camera is also required to ensure accuracy in 3D reconstruction work. In [22], the researcher applied the EM algorithm to calibrate the human action pose for multicamera linkage, and the mapping of 2D contours to 3D skeletal joints was achieved by training a neural network. Literature [23] adopts hybrid probabilistic PCA to predict the 3D body structure captured by the depth camera, which improves the 3D joint point coordinate accuracy.

**Figure 1:**
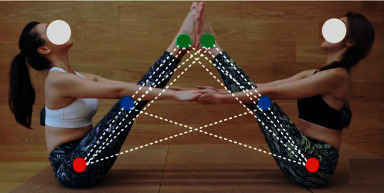
The feature communication between bone joint points.

Human motion recognition techniques originated from skeletal annotations [24, 25] by video clips [26, 27] to obtain the motion pose of each frame, which was then obtained by manual criteria. Previous human action recognition methods are based on RGB images, but this method is limited to the influence of nonobjective environments. The human skeleton-based action recognition method is less influenced by the nonobjective environment. This method can acquire the spatial-temporal features between joint points and learn the connection between features in a neural network to predict the human pose. Current neural network architectures that can be combined with the human skeleton approach are recurrent neural networks (RNN) [28, 29], long short-term memory networks (LSTM) [30, 31], convolutional neural networks (CNN) [32], etc. To make the human skeleton approach more general, [25, 26] proposed to use the heat map as a complement to the skeleton information and to use the human pose image in each video frame for the encoding process. The feature communication between bone joint points is shown in Figure 1.

**Figure 2:**
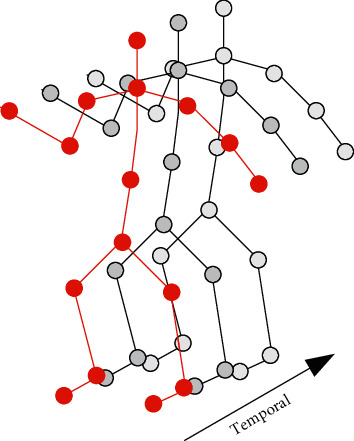
The principle of skeleton spatiotemporal feature extraction.

Literature [33] proposed a method to construct a human action dataset combining skeleton information with video in order to improve the pose estimation and action recognition accuracy of CNN networks. Literature [34] proposed a multitask parallel learning framework to improve the accuracy and stability of body joint detection. Literature [35] proposed a human intention algorithm aiming at learning behavioral action features through environmental assistance. Literature [36] took the approach of attention mechanism, which divides the human body into different parts and obtains attention from each part separately to recognize actions. Some researchers have found that the spatial-temporal graph convolution network (ST-GCN) can utilize the spatial-temporal information of skeletal articulation points effectively. It performs spatial-temporal convolution on the skeletal graph, models the graph representation of each skeleton, and uses a subsequent temporal filer to capture dynamic temporal information, as shown in Figure 2.

## 3. Method

### 3.1. Graph Convolutional Network

Benefiting from [37], the sequence of each frame *t* of the human skeleton in space is expressed as follows:

(1) $$\begin{array}{c} f_{\text{out}}=\sum_{d=0}^{D}W_{d}f_{in}\left(\Lambda_{d}^{-1/2}A_{d}\Lambda_{d}^{-1/2}⊗M_{d}\right), \end{array}$$

where *D* represents the maximum distance of the graph, *f*_*in*_ and *f*_*out*_ represent the input and output values of the feature map, ⊗ represents the multiplication function, A and *d* mark the *d*-order adjacency matrix of the joint pair, and the result of the normalization operation is represented by *A*_*d*_. *W*_*d*_ and *M*_*d*_ indicate adaptive adjustment parameters. It plays an important role in the realization of boundary adjustment and convolution operations. In order to extract temporal features, we insert a *L* × 1 convolutional layer in the shallow layer to fuse the space information of the joint points between adjacent frames. In the process of temporal feature extraction, *L* represents the length of the time window, which is a predefined hyperparameter. Each time unit and space unit are followed by a BatchNorm module and a ReLU module to form a whole with this structure.

### 3.2. Residual Unit

**Figure 3:**
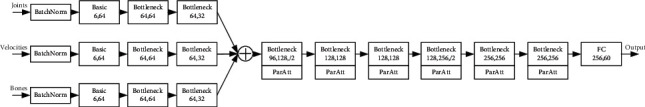
Yoga action recognition network fused with bottleneck structure.

Literature [38] proposed a structure called bottleneck, which cleverly uses the advantages of conv1×1 and is placed in the front and back positions of the common convolution part to reduce the number of feature channels in the convolution operation. In this paper, we cleverly used the bottleneck structure, abandoning the original time and space modules, and found in the experiment that the improved structure is significantly faster in model training and parameter calculation. For example, the input and output channels are 256, the channel reduction rate *r* = 4, and the time window size *L* = 9. Then, the total number of parameters involved in the calculation of the original structure is 256 × 256 × 9 = 589824. If the bottleneck structure is adopted, the total number of parameters involved in the calculation is 256 × 64 + 64 × 64 × 9 + 64 × 256 = 69632. Comparing the two, it can be seen that the bottleneck structure reduces the number of parameters calculated by the original structure by 8.5 times. Finally, we propose a new PartAtt block to enhance the generalization ability of the model. An example of a bottleneck structure frame is shown in Figure 3.

**Figure 4:**
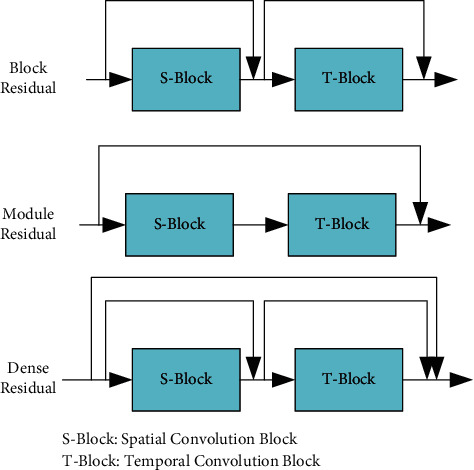
Three types of residual structure.

Considering that the time module and the space module in the original structure cannot integrate the features well, we connect the time and space modules with the residual structure to construct the ResGCN unit. The specific residual connection structure is shown in Figure 4. The Module residual module adopts a jump connection mode, the Block residual module adopts the mode of connecting before and after, the Dense residual module integrates the connection mode of the Module residual module and the Block residual module, making the structure more compact and saving calculation costs.

### 3.3. Multistream Input Structure

As we know from the bottleneck structure framework, each layer of input can be represented by a set of hyperparameters. In the first layer, we usually use basic operations to process the original input data. The second layer starts to design the bottleneck structure to filter the output data of the previous layer, and the difference in the design of the bottleneck structure is the different number of channels between the input and output. The third and fourth layers also use the bottleneck structure, but the only difference is that each layer is followed by a PartAtt unit. By introducing the PartAtt unit, all the position information of the extracted feature vector is preserved. In the decoding process, the encoding can be performed directly by the PartAtt mechanism, which reduces the intermediate steps of traditional decoding and solves the problem of feature loss. Secondly, in the PartAtt mechanism, each step of encoding and decoding directly accesses the source feature library, which realizes the direct feature tradition of encoding and decoding and shortens the exchange in feature transfer. In addition, the time step is set to 2 in the input stage of the third and fourth layers to further reduce the complexity of parameter computation and prevent overfitting problems.

Furthermore, in high-precision models, input data generally require a multistream architecture for presentation. For example, the dual-stream input architecture mentioned in [39] incorporates both joint data and skeletal data as inputs, and decision selection is made after multiple streams of inputs. This approach is adopted by most researchers because it is effective in improving model performance. However, the multistream architecture does not control the computational cost well, and the large amount is data input, parameter exchange, and variable calculation in the multistream framework, which invariably increases the huge computational volume. Therefore, our action recognition model adopts a multistream architecture in the pretraining stage, with a total of three input branches, and each input branch feature is fused with mainstream features in a pass-through tandem manner. This structure not only preserves the skeleton features to a great extent, but also makes the model more concise in its vertical structure and easier to converge when the model is trained.

In the data preprocessing stage, we mainly used the methods proposed in [29, 40] for reference. In the motion recognition method based on bone joint points, data preprocessing is very critical. In our work, preprocessing mainly revolves around joint positions, motion speeds, and bone characteristics. Suppose that a video of the action sequence is collected. According to the action sequence, the spatial coordinate set is *X*={*x* ∈ *ℝ*^*C*×*T*×*V*^}, where *C* represents the coordinates, *T* represents the frame, and *V* represents the joints. You can also get the set of relative positions of bones in space *R*={*r*_*i*_*|i*=1,2, ..., *V*}, where *r*_*i*_ = *x*[:,:, *i*] − *x*[:,:, *c*], *x* [:,: *c*] represents human bones and spinal joints. Combining the sets *R* and *X* into one set can be input into the multistream branching framework as the joint positions in action recognition. In addition, two sets of speeds of each joint can be obtained *F*={*f*_*t*_*|t*=1,2, ..., *T*} and *S*={*S*_*t*_*|t*=1,2, ..., *T*}, where *f*_*t*_ = *x* [:, *t* + 2,:] −*x*[:, *t*,:] and *S*_*t*_ = *x*[:, *t* + 1,:] − *x*[:, ]. Each motion feature of each joint can be represented by the two sets of feature vectors F and S, and this is input into the multistream branch frame as a motion stream. The basic characteristics of bones include length L={*L*_*i*_*|i*=1,2, ..., *V*} and angle A={*A*_*i*_*|i*=1,2, ..., *V*}. The angle and length of the bone can be calculated through the bone displacement relationship *l*_*i*_ = [:,:, *i*] − *x*[:,:, *i*_*adj*_ ], where the first joint of *i*_*adj*_ represents the adjacent joint. The calculation equation for the angle obtained by conversion of the customs clearance equation is as follows:

(2) $$\begin{array}{c} a_{i,w}=arccos\left(\frac{l_{i},w}{\sqrt{l_{i,x}^{2}+l_{i,y}^{2}+l_{i,z}^{2}}}\right), \end{array}$$

where *w* ∈ {*x*, *y*, *z*} represents space coordinates.

### 3.4. Partial Perception Framework

Long short-term memory neural network (LSTM) was proposed by Hochreiter [41] in 1997.

LSTM is a derivative of Recurrent Neural Network (RNN). Since 2010, it has been proven that RNN has been successfully applied to speech recognition [42], language modeling [43], and text generation [44]. However, the disappearance of gradients and explosions makes RNN difficult to apply to long-term dynamics research. As an improved network of RNN, LSTM can handle this problem well. LSTM gives the network a lot of freedom, so that the network memory unit has an adaptive solution to learn and update information, which greatly improves the performance of some perception networks.

Assume that *X*=(*x*_1_, *x*_2_, ..., *x*_*n*_) represents an input sentence composed of word representations of *n* words. In every position *t*, the RNN produces a hidden layer *h* in the middle denoted as *y*_*t*_, and the hidden state *h*_*t*_ uses a nonlinear activation function to update the previously hidden layers *h*_*t*−1_ and the input *x*_*t*_, as shown below:

(3) $$\begin{array}{c} y_{t}=\sigma \left(W_{y}h_{t}+b_{y}\right),\\ h_{t}=f\left(h_{t-1},x_{t}\right), \end{array}$$

where *W*_*y*_ and *b*_*y*_ are the parameter matrices and vectors learned during the training process, and *σ* represents the elementwise softmax function.

The LSTM unit includes an input gate *i*_*t*_, a forget gate *f*_*t*_, an output gate *o*_*t*_, and a memory unit *c*_*t*_ to update the hidden state *h*_*t*_, as shown below:

(4) $$\begin{array}{c} i_{t}=\sigma \left(W_{i}x_{t}+V_{i}h_{t-1}+b_{i}\right),\\ f_{t}=\sigma \left(W_{f}x_{t}+V_{f}h_{t-1}+b_{f}\right),\\ o_{t}=\sigma \left(W_{o}x_{t}+V_{o}h_{t-1}+b_{o}\right),\\ c_{t}=f_{t}⊙c_{t-1}+\;i_{t}⊙\;\tanh\left(W_{c}x_{t}+V_{c}h_{t-1}+b_{c}\right),\\ h_{t}=o_{t}⊙\;\tanh\left(c_{t}\right), \end{array}$$

where ⊙ is a kind of function which is similar to the multiplication operation, *V* represents a matrix related to weight, and *b* represents the learning vector. To increase the model's performance, morpheme training was carried out on two LSTMs. The first one is a morpheme that begins on the left and works its way to the right; the next one is a reverse duplicate of a character. Before passing to the next layer, the outputs of the forward and reverse passes are combined in series. Finally, the prediction value is observed using the activation function.

After understanding the partial perception algorithm LSTM, it was inspiring, because in the human body recognition process, the human skeleton will be divided into multiple parts. Each part is an interconnected joint. These parts composed of joints are made by hand, for the graph convolution to be able to explore the relationship between these parts and extract the corresponding spatial features of the joint points. To obtain the information of a point in GCN, it is necessary to start from the field of that point. According to the adjacency matrix in the field, the skeleton data is automatically segmented, and then all the feedback information is input to the next joint point to complete the capture of the feature points of the entire human skeleton. Through this operation, the defects of manual design features are avoided, and the spatial features on the time series are obtained. [45].

**Figure 5:**
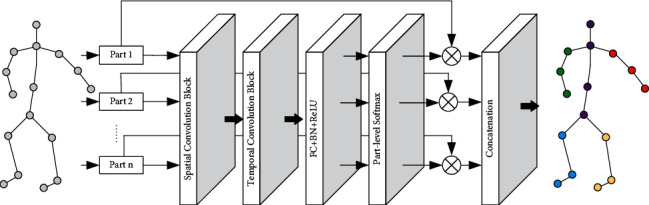
The overall network structure of yoga action recognition.

If an ordinary convolutional neural network is used, all parts will be merged into a whole for feature extraction of convolution operations. Partial perception networks can divide joints into different departments and capture individual features for each part. Separately extracting features in this way helps to explore the connection between parts, that is, the spatial-temporal relationship between joints. The structure of our proposed spatial-temporal graph convolutional network-based yoga action recognition is shown in Figure 5.

## 4. Experiments

### 4.1. Data Collection

Before the establishment of the yoga posture database, we referred to yoga courses and training materials to find a reasonable grading system to assess the risk of yoga postures. As mentioned in [46, 47], the researcher compared the physical extensibility and commonality of action between the different postures. It was also approached in terms of breathing rate, posture intensity, and meditation. Also, we interviewed a yoga instructor who showed us all the standard yoga poses and broke down each pose. From his experience's we learned that currently there are 6 main yoga poses such as standing, forward bending, sitting, twisting, back bending, and supine. Each movement determines a different level of body stretch. In the study of this paper, the grading mainly revolves around these movements; our experimental scoring is based on the depth camera directly in front as the main interface. The specific grading is shown in Table 1.

In preparing the yoga dataset, we invited a professional instructor for standard yoga posture data collection. Then we invited participants who had one year of yoga experience and those who had no previous yoga experience to divide into two groups and complete each group of movements under the guidance of the instructor. In the process of data collection, not only the body posture but also the duration and number of movements of each yoga posture were collected. The yoga duration refers to the total time from when the breathing is adjusted until after the posture is completely relaxed. The number of movements refers to the sum of all the postures done during the training period, except for some correction of the postures by the instructor. The Azure Kinect DK was used to collect yoga movement data. The data is then manually calibrated by us after the data collection is completed and the data is split. In order to enhance the validity of the data, we added confidence parameters in the coding process. The data collection results are shown in Table 2.

### 4.2. Model Training

In the model training process, we used Pytorch to implement yoga movement recognition and grading. First, we used Openpose to extract the skeleton information from the yoga video dataset, and in each frame of the video we obtained the spatial coordinate information of each of the 14 joints. Then we use the heat map as the basis for pose estimation and perform secondary feature capture on the human skeleton. Then each frame of data is arranged in the temporal dimension to correlate the features between the joints from the temporal dimension. Finally, the skeletal joint features are fused using the average prediction score and the weights are estimated in a progressive ranking. We set different learning rates at different epochs. At the beginning of training, the learning rate is set to 0.05 to adapt to the training speed of the data. Then the learning rate is set to 0.01 at epoch = 30 to speed up the learning speed; after that, the learning rate is gradually reduced at epoch = 50 and epoch = 60 to find the optimal solution. The specific parameters in the model training are shown in Table 3. All the work is done in Ubuntu 16.04 and the whole training and prediction process is done with NVIDIA TITAN *X* GPU support on Intel Xeon E5-2620 CPU.

### 4.3. Experimental Result

For the experimental data collection, we collected 50 experienced yogis and 50 inexperienced yogis. And the data was split according to the previous solution. The sensitivity, specificity, precision, and accuracy of skeletal features were captured in the data in the split starting from each frame. The experimental results are shown in Table 4. The standard yoga movements were decomposed on a larger scale, making it traceable in the validation set. Based on the above statistical results, higher sensitivity values represent more experience in yoga training and also predict closer standardization of yoga poses.

**Figure 6:**
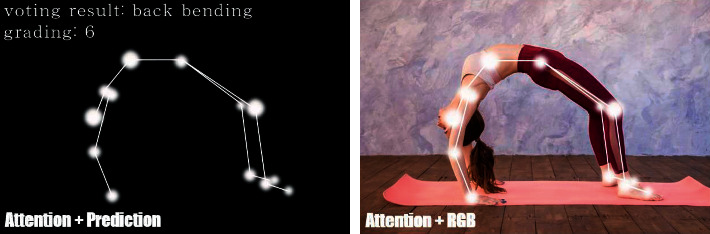
The effect of yoga action recognition and grading system.

From the above experimental results, we can see that the recognition accuracy of all yoga poses is close to 1. And the accuracies, as a kind of random error, all keep above 0.86, which proves that the model performance is still great. The gap between experienced yogis and inexperienced yogis is mainly in sensitivity and specificity. Experienced yogis scored higher in both metrics, representing the more standardized yoga poses. The yogis are captured by the depth camera while practicing yoga. Real-time skeletal joint tracking is performed on the captured video. Finally, the yoga movements are recognized with the training model and then matched with the database to generate a grading score. The specific recognition effect is shown in Figure 6.

In addition, we also made corresponding statistics in the grading, as shown in Table 5.

Table 5 demonstrates that the average grading accuracy of experienced yogis in the whole set of yoga poses is higher than that of inexperienced yogis. The yoga posture with the greatest difference was forward bending, followed by back bending. Because of the difficulty of these two poses, it was difficult for inexperienced yogis to achieve the standard poses, so the accuracy of poses grading was lower. The above experimental results favorably prove the effectiveness of the grading system in this paper, which can give yogis feedback and remind them to change their postures if the yoga movements are not standard.

## 5. Conclusion

In this paper, we found that, with the popularity of the Internet, people's lifestyles have also changed, and many people choose to learn yoga by watching videos on the Internet. For yoga beginners, learning yoga online in this way without the direct guidance of an instructor, there is a high chance that the yoga poses will be substandard. Highly difficult yoga poses are likely to be disabling for beginners. To address this potential risk, we propose a yoga posture recognition and grading system based on spatial-temporal graph convolutional neural network. We first use LSTM network to label yoga practice videos frame by frame. Then we extract the skeletal joint point features sequentially with graph convolution and then obtain the connection between joints from arranging each video frame in spatial-temporal dimension and correlating the joint points in each frame with neighboring frames for spatial-temporal information. Finally, through experiments, it is proved that our method can accurately identify yoga poses and grade them accordingly and can identify whether the yoga poses are standard or not and at the same time give feedback to yogis in a timely manner to prevent injuries to the body caused by nonstandard poses.

For deep learning algorithms, the larger the number of datasets, the better the accuracy of the model obtained from training. Since there is no specific dataset for yoga poses at present, the number of homemade datasets in this paper is small, which is the shortcoming of the work in this paper. Making datasets is a tedious and time-consuming task. In our future work, we will gradually increase the number of datasets, and at the same time, we will invest more efforts in the field of data preprocessing.

## Figures and Tables

**Table 1:** Yoga action grading details.

Posture	Grade	Frontal	Sagittal
Standing	1	8	5
Sitting	2	6	6
Supine	3	10	9
Twisting	4	6	8
Forward bending	5	7	10
Back bending	6	9	6

**Table 2:** Yoga action time and frequency.

Posture	Experienced yogis		Inexperienced yogis	
	Ave time (s)	Ave frequency	Ave time (s)	Ave frequency
Standing	15	5	10	5
Sitting	31	6	16	6
Supine	23	8	14	8
Twisting	16	6	9	6
Forward bending	32	4	11	4
Back bending	27	5	12	5

**Table 3:** Training parameter settings.

Parameter	Value
Epoch	20
Dropout rate	0.5
Initial learning rate	0.05
Learning rate (epoch = 30)	0.01
Learning rate (epoch = 50)	0.002
Learning rate (epoch = 60)	0.0004
Weight attenuation coefficient	0.0002
Momentum	0.9

**Table 4:** Yoga action recognition results.

Posture		Sensitivity	Specificity	Precision	Accuracy
Standing	Experienced yogis	0.98	0.99	0.95	0.99
	Inexperienced yogis	0.71	0.91	0.91	0.98
Sitting	Experienced yogis	0.89	0.98	0.93	0.98
	Inexperienced yogis	0.66	0.91	0.91	0.98
Supine	Experienced yogis	0.94	0.99	0.89	0.99
	Inexperienced yogis	0.78	0.93	0.91	0.97
Twisting	Experienced yogis	0.96	0.98	0.91	0.99
	Inexperienced yogis	0.77	0.94	0.89	0.99
Forward bending	Experienced yogis	0.93	0.99	0.87	0.99
	Inexperienced yogis	0.69	0.91	0.89	0.98
Back bending	Experienced yogis	0.91	0.99	0.92	0.97
	Inexperienced yogis	0.72	0.92	0.86	0.97

**Table 5:** Yoga action grading results.

Posture	Grade	Experienced yogis (%)	Inexperienced yogis (%)
Standing	1	91	80
Sitting	2	93	77
Supine	3	89	75
Twisting	4	93	76
Forward bending	5	93	71
Back bending	6	92	72
Total ave	90	75	

## Data Availability

The dataset can be accessed upon request.
